# Walkable Intelligent Parks: Can a Large Language Model Turn Urban Park Audit Findings into Actionable Recommendations?

**DOI:** 10.3390/ijerph23081035

**Published:** 2026-08-08

**Authors:** Md. Sabbir Hossain Khan, Mohammad Javad Koohsari, Jiuling Li, Jing Zhao, Yufeng Luo, Akitomo Yasunaga, Koichiro Oka, Andrew T. Kaczynski

**Affiliations:** 1 Urban Design Science for Health Laboratory, Japan Advanced Institute of Science and Technology, Nomi 923-1211, Japan; koohsari@jaist.ac.jp (M.J.K.); li-jiuling@jaist.ac.jp (J.L.); 2 Faculty of Sport Sciences, Waseda University, Tokorozawa 359-1192, Japan; koka@waseda.jp; 3 Department of Health Promotion Education and Behavior, Arnold School of Public Health, University of South Carolina, Columbia, SC 29208, USA; atkaczyn@mailbox.sc.edu; 4 School of Architecture and Urban Planning, Guangzhou University, Guangzhou 510006, China; zhaojing@gzhu.edu.cn; 5 School of Architecture, Harbin Institute of Technology, Shenzhen 518055, China; luoyf@hit.edu.cn; 6 Faculty of Health Sciences, Aomori University of Health and Welfare, Aomori 030-8505, Japan; a_yasunaga@ms.auhw.ac.jp

**Keywords:** generative artificial intelligence, human–AI comparison, environmental audit data interpretation, urban green spaces, urban design science

## Abstract

**Highlights:**

**Public health relevance—How does this work relate to a public health issue?**

- This study addresses the challenge of interpreting park audit data, which is essential for designing activity-friendly environments that support population health.
- It examines the use of a large language model (ChatGPT-5) to assist in translating audit findings into health-relevant park design insights in a dense urban context.

**Public health significance—Why is this work of significance to public health?**

- ChatGPT-5 performed comparably to experts in interpreting park audit data, indicating potential for scalable and efficient analysis.
- By generating more weakness-linked recommendations in the unadjusted analysis, the model may support preliminary synthesis of park audit findings, subject to expert review.

**Public health implications—What are the key implications or messages for practitioners, policy makers and/or researchers in public health?**

- Large language models can support evidence-based park improvements to promote physical activity and public health but still require expert oversight for contextual feasibility and local relevance.

**Abstract:**

Park audits can inform neighbourhood-scale urban design strategies that support health. However, interpreting park audit data is time-consuming and requires specialised expertise. Recent advances in large language models suggest potential for assisting with structured data interpretation, but their application to park audits remains unexplored. This study examined whether a large language model (ChatGPT-5) can assist in interpreting park audit data from public parks in Dhaka City, Bangladesh. Using a modified park audit tool, 59 parks were audited, of which 26 met the predefined completeness threshold and were included in the analysis. ChatGPT-5 and human experts received identical park audit datasets and instructions for interpretation. Outputs were evaluated using content analysis and a pre-defined scoring framework to assess accuracy and comprehensiveness. Paired *t*-tests compared ChatGPT-5 outputs with the expert benchmark. ChatGPT-5 showed no statistically significant difference from the expert benchmark in overall accuracy in this sample (*p* = 0.13). The unadjusted analysis showed a higher recommendation-match count for ChatGPT-5 (*p* < 0.05), but this result should be interpreted as exploratory because multiple criteria were tested. No statistically significant differences were detected across accuracy domains or other comprehensiveness criteria. These findings suggest that ChatGPT-5 may assist with preliminary interpretation of structured park audit data. However, the results do not establish equivalence with expert assessment. Expert review remains necessary to assess feasibility, contextual relevance, and alignment with local planning standards.

## 1. Introduction

Built environment elements such as street patterns, density, and land use influence opportunities for physical activity and related health outcomes [1,2]. Of these various neighbourhood attributes, public open spaces, particularly urban parks, provide accessible spaces for walking and other active behaviours [3]. Several studies have found that park proximity, facility diversity, and the provision of open space in the park are associated with residents’ greater physical activity [4,5,6]. Further, certain park features, such as walking paths and bike tracks, can support active park use [5,7]. However, in dense urban settings, finite land area and the high competition for space can limit the facilities and features of parks [8,9]. These limitations indicate a need for park (re)design to increase opportunities for walking in dense urban settings where land for green space is in short supply.

Improving park quality requires a systematic assessment of existing conditions. Previous studies have assessed park environments using perceived measures reported by users or objective measures based on direct observation or spatial data [10]. Audit tools are a major objective approach because they directly record park environments in a standardised way [11]. However, park audit outputs are not straightforward to translate into actionable guidance. Although numerous audit tools have been developed to evaluate park environments [9,12,13,14,15], interpreting audit data remains challenging. Typically, a park audit tool consists of many isolated items that individually assess a particular feature [16]. Users must synthesise these item-level observations into an overall interpretation and then develop (re)design recommendations. This process relies on professional judgement, and it is highly cognitively demanding. It may also require repeated cross-checking across park items and domains, which can slow decision-making. Additionally, the intricacy and diversity of audit items, evaluator judgement variation, and different contexts of urban settings are some factors that serve as constraints to comparability [16,17]. Furthermore, park audit data interpretation becomes more difficult with limited resources or funding [18,19]. Most of the park audit literature has concentrated on instrument development, while the post-collection stage (e.g., synthesising, interpreting, and formulating recommendations) has been given little consideration. Evidence from more mature fields shows that comprehensive audit processes involve interpretation and reporting with recommendations [20,21]. These stages require specialised knowledge and are resource-intensive when undertaken manually. Efficient, scalable, and reliable approaches for interpreting park audit data are therefore needed.

Large language models (LLMs) are transformer-based systems and are trained on large text data to generate context-sensitive outputs in response to prompts [22,23]. LLMs have been used to assist with data interpretation, knowledge synthesis, and decision support across diverse fields [24,25]. For example, work in software engineering suggests that LLM-as-a-judge approaches can apply rule-based criteria with near-human performance in structured evaluation tasks [26]. Further supportive evidence from research on unstructured text also shows that LLMs can consistently apply a given set of rules across different inputs, which is important for structured interpretation tasks [27]. This capability is relevant to park audit contexts because audit tools usually produce many item-level observations that require integration across domains. Previous studies have shown that LLMs can perform at levels comparable to or exceeding those of human experts in clinical, educational, and urban planning tasks [28,29,30]. These studies suggest that LLMs have considerable potential for expert-assisted interpretation, knowledge synthesis, and recommendation generation across diverse domains. This represents an important knowledge gap, given the growing reliance on park audits to inform planning and public health decision-making. However, LLM applications for interpreting park audit data and generating park (re)design recommendations have not been examined to date.

Therefore, this study evaluated whether an LLM can interpret structured park audit data and generate park (re)design recommendations in a high-density urban context. Dhaka City Corporation in Bangladesh was selected because it is among the most densely populated urban areas globally [31,32]. The audiT tool for Activity-friendly Parks in denSe urban areas (TAPS) was used, as it was specifically developed for dense urban settings [9]. ChatGPT-5 (default model, free web version; OpenAI, San Francisco, CA, USA) was evaluated as the tested LLM, and its outputs were compared with an expert benchmark to assess accuracy and the alignment of recommendations with audited weaknesses.

The remainder of this paper is organised as follows. Section 2 describes the study design, park audit procedures, interpretation protocol, scoring framework, and statistical analyses. Section 3 presents the results. Section 4 discusses the findings, implications, strengths, and limitations of the study. Section 5 concludes the paper and outlines directions for future research.

## 2. Materials and Methods

### 2.1. Study Design

The study used a comparative evaluation design that combined qualitative content coding with quantitative scoring. The aim was to compare how human experts and an LLM (ChatGPT-5) interpret structured park audit data. However, ChatGPT is one of the most widely adopted LLMs. It uses transformer-based systems to handle vast data efficiently. As a result, it shows strong performance in both understanding and generating text [33]. Moreover, ChatGPT is freely accessible and easy to use, making it a cost-effective tool for a wide range of users [34]. Therefore, ChatGPT-5 was selected as a representative state-of-the-art large language model to evaluate the feasibility of LLM-assisted interpretation of structured park audit findings. Additionally, the study was designed as a feasibility assessment rather than a comparative benchmarking exercise across multiple LLMs. Accordingly, a single state-of-the-art LLM (ChatGPT-5) was selected to examine whether structured park audit findings could be interpreted and translated into recommendations comparable to those generated by experts. This approach is consistent with previous studies that have evaluated the performance of a single LLM against expert-generated responses within a defined application context [29,35]. On the other hand, park audit data were collected using a modified version of the TAPS tool, adapted to the Dhaka City Corporation context in Bangladesh. An identical standardised prompt was used to obtain interpretations from both human experts and ChatGPT-5. Outputs were evaluated through two complementary approaches. First, content analysis was employed to code predefined categories informed by the TAPS framework [36,37]. This analysis assessed interpretation characteristics across seven comprehensiveness criteria: domain coverage, user coverage, recommendations match, recommendations mismatch, strengths count, weaknesses count, and issue resolution rate. Second, a structured scoring approach assessed the accuracy of descriptive summaries. Accuracy was defined as the degree to which the statements in each summary matched the corresponding park audit data. A numerical codebook specified how domain-level scores were calculated from audited items and supported consistent scoring across domains. These domain-level scores were then used to evaluate the accuracy of audit interpretations. Ethical approval was not required because the study did not collect identifiable personal data.

### 2.2. Park Audit Data Collection

The study was conducted in Dhaka City Corporation, which is among the most densely populated areas in the world [31,32]. Park environments were assessed using the TAPS, which was developed for dense urban settings. It comprises 74 items across 24 criteria and five domains (surroundings and accessibility, activity area, facilities and amenities, aesthetics, and safety). The instrument was designed to provide a standardised assessment of park characteristics that support physical activity and active park use in high-density urban environments. Selected TAPS items were adapted to represent the local context. For example, “soccer” was replaced with “football”. “Softball” was removed from the activity area domain, and “basketball” was added to reflect a common local activity. The sequence and selection of sports-court items were also adjusted to reflect locally common recreational facilities, including prioritising cricket-related items.

Official inventories listed 27 parks in Dhaka South City Corporation and 18 parks in Dhaka North City Corporation. Field surveys identified 14 additional publicly accessible parks not included in these inventories. All identified parks were included, yielding 59 parks for auditing. Data were collected by three trained auditors who divided the parks across the team. Auditors completed an online training session on 17 April 2025, led by the TAPS developer (YL), followed by a pilot audit in a park outside Dhaka City Corporation. A follow-up meeting was held on 11 May 2025 to resolve questions and align scoring procedures with developer guidance. The main audits were collected from 28 May to 5 June 2025 across all the 59 parks. Auditors received BDT 1000 per park audited.

Each park audit dataset was completed for the 74 TAPS items. Parks were then classified by the percentage of items recorded, which was used as an indicator of audit completeness. In total, 12 parks had ≥50% item coverage, 26 had ≥40%, and 35 had ≥30%, with the lowest coverage at 4.05%. A minimum threshold of 40% item coverage was predefined as a study-specific operational criterion to ensure sufficient information for meaningful interpretation. Because the objective of this study was to evaluate interpretation performance rather than estimate park quality, parks with substantially lower completion levels frequently lacked information across multiple audit domains, increasing interpretive uncertainty. Accordingly, 26 parks were included in the subsequent interpretation, and the corresponding datasets were prepared for the present analysis.

### 2.3. Interpretation Procedures for Experts and ChatGPT-5

#### 2.3.1. Materials Prepared for Interpretation

Audit data from the 26 included parks were compiled into park-specific datasets. Each dataset listed the 74 TAPS items and the recorded response for that park. The datasets were provided as Microsoft Word documents (Microsoft Corporation, Redmond, WA, USA), and a single prompt was also provided. The prompt instructed the reviewers to produce a summary of park-identifying strengths and weaknesses and proposing recommendations to enhance the park’s capacity to support physical activity. The same dataset format and prompt were used for both experts and ChatGPT-5 interpretation to ensure comparability. The prompt stated:

“We are providing the audit data for a park located in a densely populated area. Based only on this information, please:

1. Write a short summary within 50–70 words highlighting the park’s strengths and weaknesses in promoting physical activity.
2. Based on the above summary, please recommend two specific physical design interventions to enhance the park’s capacity to support physical activity.”

The same prompt was used for all parks, and no follow-up prompts, clarifications, or external tools were used. In subsequent coding, individual actionable components within each intervention were counted separately when they addressed distinct weaknesses. An example dataset with this prompt is provided in Supplementary Materials S1.

#### 2.3.2. Expert Interpretation Procedure

Two experts from the author team independently interpreted the 26 park datasets and served as the expert benchmark. One expert had experience in developing and applying park audit tools (TAPS), and another had experience conducting park and public space audits with related academic background. Their role was limited to producing expert interpretations. They did not score the accuracy of responses or conduct the content coding used for the comparative analysis. Park identities were masked and parks were labelled using study IDs. Experts worked independently and did not use external references or generative artificial intelligence (AI) tools. Furthermore, both experts completed their interpretations independently, received identical datasets and standardised instructions, and remained blinded to ChatGPT-5 outputs throughout the interpretation process. Before the main task, both experts jointly interpreted two hypothetical park datasets that were not part of the study. This step was used to align interpretation expectations related to the prompt, output length, and the level of specificity. Each expert produced one interpretation per park (52 expert interpretations in total).

#### 2.3.3. ChatGPT-5 Interpretation Procedure

The ChatGPT-5 model was accessed through the ChatGPT interface (OpenAI, L.L.C., San Francisco, CA, USA). For each of the 26 parks, the park-specific dataset was entered into ChatGPT-5 on 4 October 2025. Each park was interpreted in a separate new chat session using the same standardised prompt. Memory was switched off and no additional tools or plugins were enabled to minimise carryover between parks [35]. All ChatGPT-5 outputs were recorded in their original form, producing a total of 26 unedited interpretations.

### 2.4. Content Analysis

Content analysis was used to characterise and compare the interpretive content of expert and ChatGPT-5 outputs. All responses were imported into NVivo version 15 (Lumivero, Denver, CO, USA) for coding. A deductive coding framework was applied using predefined categories. The framework focused on counts and alignments that could be derived directly from the written outputs. Each park interpretation was coded for the following criteria: strengths count, weaknesses count, recommendations match, recommendations mismatch, user coverage, domain coverage, and issue resolution rate. Strengths count was defined as the number of distinct positive park attributes stated in the summary. Each attribute was counted once. Weaknesses count was defined as the number of negative or deficient attributes stated in the summary, with each attribute counted once. Recommendations match referred to the number of distinct actionable recommendation components that directly addressed the weaknesses mentioned in the summary. Although the prompt requested two physical design interventions, each intervention could contain more than one actionable component. For example, one intervention could include adding shaded walking paths, improving lighting, and installing seating. Each distinct component was counted separately when it directly addressed a stated weakness. A recommendation component was coded as a match only when a clear linkage to a specific weakness was evident. The same coding rule was applied to expert and ChatGPT-5 outputs. Recommendations mismatch was defined as the number of recommendations that did not address any stated weaknesses in the summary. These included suggestions that were general or unrelated to the weaknesses identified. User coverage referred to the number of distinct user groups mentioned in the interpretation. Each group was counted once (e.g., children, adults, and older adults). This measure captured the diversity of park users acknowledged in the interpretation. Domain coverage referred to the number of distinct TAPS domains (out of 5) addressed within the summary. An issue resolution rate was computed as recommendations match divided by the weaknesses count. This content analysis was conducted by one coder (JL) using the predefined coding framework. It provided standardised quantitative descriptors of the expert and ChatGPT-5 interpretations. Because the content coding was conducted by one coder, potential coder subjectivity is acknowledged as a limitation.

### 2.5. Structured Scoring

A structured scoring framework was established to evaluate the accuracy of park-level summaries produced by ChatGPT-5 and the two experts. A numerical codebook was developed based on the TAPS item structure to guide domain-level score calculation and standardise scoring across domains. The codebook specified the steps required to compute each domain score from relevant audit items. Using this codebook, domain-level scores were calculated for each of the 26 included parks. Two independent raters (JZ and JL) evaluated the accuracy of all 78 responses (52 expert responses and 26 ChatGPT-5 responses) using a three-point scale (1 = accurate, 0.5 = partially accurate, and 0 = inaccurate). Raters were blinded to the resource source. Accuracy assessment was restricted to the descriptive summary section of each response. For item-level statements (for example, “this park has no rubbish bins”), raters compared the statement with the corresponding TAPS item response to determine accuracy. For broader evaluative statements (for example, “this park is safe for residents”), raters assessed accuracy by comparing the statement with the relevant domain-level score derived from the codebook. The same three-point scale was applied across both item-level and domain-level assessments. The scoring structure and illustrative examples are provided in Supplementary Materials S2. Raters completed assessments independently. Disagreements at the item level were discussed to reach consensus, and 11 unresolved cases were adjudicated by a third reviewer (MJK). The analysis was based on final consensus ratings following independent review and adjudication. The original independent scoring matrices were not retained in a format suitable for calculating formal inter-rater reliability statistics. Therefore, inter-rater reliability could not be reported. This is acknowledged as a methodological limitation.

### 2.6. Statistical Analysis

The park was the unit of analysis (*n* = 26). For each park, domain-level interpretation scores from two experts were averaged to derive a single expert benchmark value for each criterion. Descriptive statistics were calculated to summarise the distribution of scores for ChatGPT-5 and the expert benchmark. Paired samples *t*-tests were applied to examine mean differences between ChatGPT-5 and expert benchmark values across criteria. To assess the robustness of the findings given the ordinal nature of the underlying accuracy ratings, supplementary analyses were also conducted using the Wilcoxon signed-rank test. Statistical significance was set at *p* < 0.05. Because multiple criteria were tested in this feasibility study, *p* values were interpreted cautiously and the analyses were treated as exploratory rather than confirmatory. All analyses were conducted using IBM SPSS Statistics version 30 (IBM Corp., Armonk, NY, USA).

## 3. Results

Table 1 presents the descriptive comparison of ChatGPT-5 and expert evaluation scores across accuracy and comprehensiveness criteria. For the accuracy-related measures, the experts reported higher mean values in safety, surroundings and accessibility, facilities and amenities, and overall accuracy. In safety, experts scored 0.96 (SD = 0.09, CV = 0.10) compared with ChatGPT-5′s 0.79 (SD = 0.36, CV = 0.45). For surroundings and accessibility, experts achieved 0.96 (SD = 0.14, CV = 0.14), whereas ChatGPT-5 scored 0.88 (SD = 0.21, CV = 0.24). Similarly, in facilities and amenities, expert scores were 0.83 (SD = 0.21, CV = 0.25) and ChatGPT-5 scored 0.81 (SD = 0.29, CV = 0.35). For overall accuracy, experts obtained 0.92 (SD = 0.08, CV = 0.08) compared with ChatGPT-5′s 0.88 (SD = 0.12, CV = 0.14). In contrast, ChatGPT-5 reported higher mean values only in the activity area, with a mean score of 0.98 (SD = 0.10, CV = 0.10) compared with 0.93 (SD = 0.11, CV = 0.12) for the expert assessment. Aesthetics was the only criterion for which the mean values were equal for both assessors. ChatGPT-5 reported 0.92 (SD = 0.19, CV = 0.21), whereas the expert scored 0.92 (SD = 0.18, CV = 0.20).

Across the seven comprehensiveness-related criteria, the performance pattern was more evenly divided. ChatGPT-5 reported higher mean values in domain coverage, recommendations match, and issue resolution rate. In domain coverage, ChatGPT-5 recorded 4.12 (SD = 0.86, CV = 0.21), whereas experts scored 3.90 (SD = 0.74, CV = 0.19). For recommendations match, ChatGPT-5 scored 5.12 (SD = 1.40, CV = 0.27) compared with the experts’ 4.50 (SD = 1.17, CV = 0.26). In issue resolution rate, ChatGPT-5 achieved 0.73 (SD = 0.18, CV = 0.25), whereas experts reported 0.66 (SD = 0.11, CV = 0.17). Three criteria exhibited closely aligned performance between the two types of evaluators. In strengths count, ChatGPT-5 recorded 5.46 (SD = 2.12, CV = 0.39) and experts reported 5.60 (SD = 1.59, CV = 0.28). For weaknesses count, ChatGPT-5 scored 7.08 (SD = 1.44, CV = 0.20) and experts scored 7.33 (SD = 1.76, CV = 0.24). In user coverage, ChatGPT-5 produced 1.50 (SD = 0.65, CV = 0.43) while experts reported 1.58 (SD = 0.66, CV = 0.42). The remaining comprehensiveness criterion, recommendations mismatch, showed low mean values but substantial variability for both types of evaluators. ChatGPT-5 reported 0.92 (SD = 0.98, CV = 1.06), whereas experts recorded 0.63 (SD = 0.64, CV = 1.01), reflecting the highest coefficients of variation within the dataset for both sources.

As shown in Table 2, the unadjusted paired samples *t*-tests indicated that ChatGPT-5 generated a higher recommendations-match score than the experts, with a mean difference of 0.62 (95% CI = 0.01–1.22; *p* < 0.05). This result should be interpreted as exploratory because 13 criteria were tested and no multiple-comparison correction was applied. The remaining criteria, including surroundings and accessibility, activity area, facilities and amenities, aesthetics, safety, overall accuracy, domain coverage, strengths count, weaknesses count, recommendations mismatch, user coverage, and issue resolution rate showed no statistically significant differences between ChatGPT-5 and expert evaluations. Additional Wilcoxon signed-rank tests yielded a similar pattern in the unadjusted sensitivity analysis, with recommendations match being the only criterion with *p* < 0.05, while all other comparisons remained non-significant (Supplementary Materials S3). This sensitivity analysis was also exploratory.

## 4. Discussion

This study examined whether an LLM (ChatGPT-5) can interpret structured park audit outputs at the level of expert assessors. Overall, no statistically significant difference was detected between ChatGPT-5 and the expert benchmark for most evaluative criteria in this sample. This should not be interpreted as evidence of statistical equivalence or non-inferiority. To our knowledge, no prior study has compared LLM and expert interpretations of park audit data. However, the observed alignment is consistent with studies in other fields, including clinical and public health applications of LLMs for text classification and evidence summarisation [38,39,40]. This study extends this evidence to park auditing within a rule-based audit framework. This is relevant for high-density cities, where limited land increases the demand for efficient and timely park audit interpretation to support park improvement decisions.

Recommendations match was the only criterion with *p* < 0.05 in the unadjusted analyses. In interpreting park audits, ChatGPT-5 generated more distinct recommendation components per park that directly addressed a weakness stated in its park summary. However, this finding should be interpreted as exploratory because multiple criteria were tested and no multiple-comparison correction was applied. This pattern is plausible given the controlled task conditions in this study. Each park dataset presented itemised audit responses, and task instructions were the same for both sources. Under fixed inputs and rules, an LLM can apply a clear mapping from stated weaknesses to corresponding actions [41]. A related interpretation issue is that strong linkages can occur even when a summary is not fully faithful to the related park audit items. However, the accuracy-related criteria showed no significant differences between ChatGPT-5 and the expert benchmark. Expert raters may also add contextual considerations (e.g., cultural and political issues, expertise bias, personal preferences), which may result in suggestions being less related to the park’s listed weaknesses [42]. This finding aligns with evidence that LLMs can match or exceed human performance on tightly specified judgment and coding tasks, when decision rules are clearly structured [26,43,44]. For research and practice focused on activity-friendly neighbourhoods, the implication is that LLMs may help translate park audit deficits into activity-relevant design actions. However, the practical implementation of these recommendations may depend on contextual factors such as available resources, governance arrangements, land availability, and local planning priorities. Consequently, the recommendations should be viewed as preliminary decision-support outputs rather than ready-to-implement planning solutions. Although the study was conducted in Dhaka, the underlying approach may be transferable to other settings where standardised park audit tools are used, provided that local contextual factors are considered during interpretation and implementation.

Across the other evaluation criteria, no statistically significant differences were detected between ChatGPT-5 and the expert benchmark. These results indicate that the study did not detect clear differences under the present sample size and task conditions. They should not be interpreted as evidence that ChatGPT-5 and expert interpretations were equivalent. Most domains consisted of itemised and closed-response items that capture observable park attributes, including surroundings and accessibility, activity areas, facilities and amenities, aesthetics, and safety. Such audit structures reduce interpretative discretion and reduce variability between interpreters [45]. The same logic also applies to count-based measures, such as strengths count, weaknesses count, and user coverage, which depend on the clarity of the statements. Composite indices, including overall accuracy and issue resolution rate, combine information across items and domains and thus may not be very sensitive to slight differences in emphasis. These results imply that, with the condition of standardising audit inputs and prompts, LLM-generated outputs can approximate expert benchmark scores for describing audited park attributes. This can be a basis for multi-park comparisons in studies of activity-friendly parks and neighbourhoods. This may also accelerate park improvement processes by enabling planning staff to focus on higher-level tasks, such as prioritisation, contextual evaluation, and implementation planning. However, the findings should be interpreted with caution because different prompt configurations may produce different outputs, which were not evaluated in the present study. Among the five domains, the safety domain showed the lowest mean accuracy and highest variability for ChatGPT-5, whereas expert evaluations remained consistently high. One possible explanation is that safety-related interpretations may require domain-specific practical knowledge of park design and use. Experts may have been better able to synthesise these factors when interpreting the structured audit data, whereas ChatGPT-5 showed greater variability in its interpretation of safety-related characteristics. The findings suggest potential utility for supporting the translation of park audit findings into preliminary improvement recommendations. However, validation across different geographical contexts and audit instruments is required before broader implementation can be considered.

This study has several limitations. First, the analysis was conducted using only one LLM, and the performance of different models, versions, or updates in the future may vary. In addition, only a single standardised prompt was used, and prompt sensitivity was not evaluated. Different prompt formulations may produce different interpretations and recommendations. Second, the 40% completeness threshold was adopted as a study-specific operational criterion to ensure sufficient audit information for interpretation and should not be considered a universally established standard. Third, the evaluation was limited to the interpretation of the audit data and the generation of textual summaries and recommendations. In addition, output stability across repeated ChatGPT-5 runs was not assessed. Nevertheless, previous studies have reported relatively consistent performance of LLMs under standardised prompting conditions [35,46]. The study did not check the feasibility, implementation, or effectiveness of the proposed actions in real parks. Furthermore, formal inter-rater reliability statistics could not be calculated because the original independent scoring matrices were not retained in a format suitable for reliability analysis. This is a methodological limitation because the degree of initial agreement among raters cannot be quantified. In addition, the expert benchmark was generated by two coauthors, which may introduce potential expert-benchmark bias despite independent interpretation, masked park identities, and blinding to ChatGPT-5 outputs. The content analysis was also conducted by one coder, which may introduce coder subjectivity. Future studies should retain independent scoring matrices, report inter-rater reliability, include external expert benchmarks, and use duplicate coding for content analysis. Finally, the study examined only one park audit instrument, which does not permit direct comparison with other park audit tools. The study also has several strengths. Using the same structured audited data and the same standardised prompt enabled a controlled comparison between ChatGPT-5 and expert interpretations. This minimised interpretive discretion in domains grounded in observable attributes and closed-response items. The evaluation also combined accuracy-related scores with content-based criteria, which allowed a comprehensive examination of LLM-expert alignment within a rule-based audit framework.

## 5. Conclusions

This study provides initial evidence from a single-city case study on how an LLM (ChatGPT-5) interprets structured park audit outputs relative to expert benchmarks. In this single-city feasibility study, no statistically significant difference was detected between ChatGPT-5 and experts for most evaluated criteria. This finding should be interpreted cautiously because the study was not designed as an equivalence or non-inferiority analysis. These findings suggest that, within a rule-based audit framework, LLM (ChatGPT-5)-assisted interpretations may support scalable synthesis of audited park attributes and weakness-linked improvement actions for walking. Expert judgement is still required to determine the feasibility of the recommendations for park improvements and the application of local standards. Future studies should test across different cities, countries, and audit contexts and whether LLM-generated recommendations lead to actionable park improvements and measurable park-based walking outcomes.

## Figures and Tables

**Table 1:** Descriptive Comparison of ChatGPT-5 and Expert Evaluation Scores for 26 Audited Parks (*n* = 26).

Criteria	Source	Minimum	Maximum	Mean (SD ^b^, CV ^c^)
Accuracy in park surroundings and accessibility	ChatGPT-5	0.50	1.00	0.88 (0.21, 0.24)
	Expert	0.50	1.00	0.96 (0.14, 0.14)
Accuracy in activity area	ChatGPT-5	0.50	1.00	0.98 (0.10, 0.10)
	Expert	0.75	1.00	0.93 (0.11, 0.12)
Accuracy in facilities and amenities	ChatGPT-5	0.00	1.00	0.81 (0.29, 0.35)
	Expert	0.50	1.00	0.83 (0.21, 0.25)
Accuracy in aesthetics	ChatGPT-5	0.50	1.00	0.92 (0.19, 0.21)
	Expert	0.50	1.00	0.92 (0.18, 0.20)
Accuracy in safety	ChatGPT-5	0.00	1.00	0.79 (0.36, 0.45)
	Expert	0.75	1.00	0.96 (0.09, 0.10)
Overall accuracy ^a^	ChatGPT-5	0.63	1.00	0.88 (0.12, 0.14)
	Expert	0.75	1.00	0.92 (0.08, 0.08)
Domain coverage	ChatGPT-5	3.00	5.00	4.12 (0.86, 0.21)
	Expert	2.00	5.00	3.90 (0.74, 0.19)
Strengths count	ChatGPT-5	1.00	10.00	5.46 (2.12, 0.39)
	Expert	2.00	8.00	5.60 (1.59, 0.28)
Weaknesses count	ChatGPT-5	5.00	10.00	7.08 (1.44, 0.20)
	Expert	4.50	11.50	7.33 (1.76, 0.24)
Recommendations match	ChatGPT-5	3.00	8.00	5.12 (1.40, 0.27)
	Expert	2.00	7.50	4.50 (1.17, 0.26)
Recommendations mismatch	ChatGPT-5	0.00	4.00	0.92 (0.98, 1.06)
	Expert	0.00	2.50	0.63 (0.64, 1.01)
User coverage	ChatGPT-5	0.00	3.00	1.50 (0.65, 0.43)
	Expert	0.50	3.00	1.58 (0.66, 0.42)
Issue resolution rate	ChatGPT-5	0.50	1.00	0.73 (0.18, 0.25)
	Expert	0.44	0.92	0.66 (0.11, 0.17)

^a^ Overall accuracy represents the average accuracy score across the five park domains; ^b^ SD: standard deviation; ^c^ CV: coefficient of variation. Recommendations-match and recommendations-mismatch values refer to distinct actionable recommendation components, not to the number of intervention paragraphs requested in the prompt.

**Table 2:** Comparison of ChatGPT-5 and Expert Scores Using Paired Samples *t*-tests.

Criteria	Group	Paired Differences			t	df	Sig. (Two-Tailed)
		Mean (SD ^d^)	Std. Error Mean	95% CI ^e^ of the Difference			
Accuracy in park surroundings and accessibility	ChatGPT-5—Expert	−0.08 (0.23)	0.05	−0.17–0.02	−1.69	25	0.10
Accuracy in activity area	ChatGPT-5—Expert	0.05 (0.12)	0.02	0.00–0.10	2.00	25	0.06
Accuracy in facilities and amenities	ChatGPT-5—Expert	−0.02 (0.32)	0.06	−0.15–0.11	−0.30	25	0.76
Accuracy in aesthetics	ChatGPT-5—Expert	−0.02 (0.18)	0.05	−0.14–0.09	−0.43	10 ^a^	0.68
Accuracy in safety	ChatGPT-5—Expert	−0.19 (0.40)	0.11	−0.43–0.05	−1.75	12 ^b^	0.11
Accuracy ^c^	ChatGPT-5—Expert	−0.04 (0.13)	0.03	−0.09–0.01	−1.55	25	0.13
Domain coverage	ChatGPT-5—Expert	0.21 (1.07)	0.21	−0.22–0.64	1.01	25	0.32
Strengths count	ChatGPT-5—Expert	−0.13 (2.67)	0.52	−1.21–0.94	−0.26	25	0.80
Weaknesses count	ChatGPT-5—Expert	−0.25 (1.48)	0.29	−0.85–0.35	−0.86	25	0.40
Recommendations match	ChatGPT-5—Expert	0.62 (1.50)	0.29	0.01–1.22	2.09	25	*p* < 0.05 *
Recommendations mismatch	ChatGPT-5—Expert	0.29 (1.19)	0.23	−0.19–0.77	1.23	25	0.23
User coverage	ChatGPT-5—Expert	−0.08 (1.12)	0.22	−0.53–0.38	−0.35	25	0.73
Issue resolution rate	ChatGPT-5—Expert	0.07 (0.19)	0.04	−0.01–0.15	1.89	25	0.07

^a,b^ Degrees of freedom vary because some parks could not receive a domain-level score in the aesthetics and safety domains; ^c^ accuracy represents the average accuracy score across the five park domains; ^d^ SD: standard deviation; ^e^ CI: confidence interval; * *p* < 0.05.

## Data Availability

The materials required to reproduce the analysis are available in the Supplementary Materials. These materials include the park audit datasets used for analysis, the exact prompt, ChatGPT-5 outputs, expert outputs, the scoring codebook, final consensus ratings, content-coding outputs, and Wilcoxon signed-rank results. No identifiable personal data were collected.

## Supplementary Materials

The following supporting information can be downloaded at https://www.mdpi.com/article/10.3390/ijerph23081035/s1. Supplementary Materials S1: Prompt for the experts and ChatGPT-5 and an example dataset for Park A; Supplementary Materials S2: Numerical codebook derived from the TAPS items structure to guide domain-level score calculation; Supplementary Materials S3: Comparison of ChatGPT-5 and Expert Scores Using Wilcoxon Signed-Rank Test.
