# Supplementary material for: Walkable Intelligent Parks: Can a Large Language Model Turn Urban Park Audit Findings into Actionable Recommendations?
Source: Int J Environ Res Public Health. 2026 Aug 8;23(8):1035. doi: 10.3390/ijerph23081035 (PMC13512454; doi:10.3390/ijerph23081035)
Supplement: Supplementary file 1 [file ijerph-23-01035-s001.zip › ijerph-4304242-supplementary.pdf]

Supplementary Materials S1: Prompt for the experts and ChatGPT-5 and an example dataset for Park A

Prompt for the Experts and ChatGPT-5 Response

"We are providing the audit data for a park located in a densely populated area. Based only on this information, please:

1. Write a short summary within 50-70 words highlighting the park's strengths and weaknesses in promoting physical activity.
2. Based on the above summary, please recommend two specific physical design interventions to enhance the park's capacity to support physical activity."

Park name: Park A

Park surroundings and accessibility

Entrance points and signage

1. How many entrance points are there to access the park smoothly?

Answer – "3-5"

2. Are there any signs showing the location or directions to the park?

Answer – "No"

3. Is there any park information at the entrance points? (e.g., park name, maps, opening hours, rules and prohibitions)

Answer – "Yes"

4. Are the entrance points attractive for residents?

Answer – "Some"

Traffic safety

5. Are there any crosswalks on the road to access the park?

Answer – "Yes"

6. Are there any traffic lights on the crosswalk?

Answer – "No"

7. Are there any signs of a speed limit on the roads to access the park?

Answer – "No"

8. Is the traffic situation around the park generally safe for residents?
Answer – “A lot”

Roads bordering the park

9. Are there sidewalks/bike lanes on the roads bordering the park?
Answer – “Yes”
10. Is the width of the sidewalk/bike lane suitable for walking/cycling?
Answer – “No”
11. Are there any obstacles on the sidewalk/bike lane that prevent you from moving on?
Answer – “Yes”

Public transport station

12. Are there any public transport stations close to the park? (Please use the “Google Map” to search for all public stations within 15 min walk to the park. Please check the distance from every entrance point of the park while searching in Google Map)
Answer – “Yes”
13. How long is the walking or cycling time from the public transport station to the nearest park entrance point (min)?
Answer – “6-10”

Parking lot

14. Are there any parking lots? (Specifically provided by the park.) (If yes, how many parking spaces are there?)
Answer – “Yes, 20 parking spaces”
15. Are there any bicycle racks? (Specifically provided by the park.) (If yes, how many bicycle racks are there?)
Answer – “No”
16. Is there any parking fee?
Answer – “No”
17. Is it convenient for parking? (Consider the entrance and exit signs, and parking management）
Answer – “A lot”

Activity area

Sport court

18. What sports courts are in the park?
Answer – “Cricket pitch, Football ground, and Basketball court”
19. Is the cricket pitch usable?
Answer – “Yes”
20. Is the cricket pitch free to use?
Answer – “Yes”
21. Are there any guidance or warning signs for cricket pitch users?
Answer – “Yes”
22. Are there any benches to rest on?
Answer – “Yes”
23. Are there any shade areas or shelter in the cricket pitch users?
Answer – “Yes”
24. Is the cricket playing area fenced?
Answer – “Yes”
25. Is the cricket pitch in good condition?
Answer – “Some”
26. Is the football ground usable?
Answer – “Yes”
27. Is the football ground free to use?
Answer – “Yes”
28. Are there any guidance or warning signs for football ground users?
Answer – “Yes”
29. Are there any benches to rest on?
Answer – “Yes”
30. Are there any shade areas or shelter in the football ground users?
Answer – “No”
31. Is the football playing area fenced?
Answer – “Yes”
32. Is the football ground in good condition?
Answer – “A lot”
33. Is the basketball court usable?
Answer – “Yes”
34. Is the basketball court free to use?
Answer – “Yes”
35. Are there any guidance or warning signs for basketball court users?
Answer – “No”
36. Are there any benches to rest on?
Answer – “No”

37. Are there any shade areas or shelter in the basketball court users?
Answer – “No”
38. Is the basketball playing area fenced?
Answer – “Yes”
39. Is the basketball court in good condition?
Answer – “A lot”

Kid’s playground

40. Are there any Kid’s playgrounds in the park?
Answer – “Yes”
41. Are the Kid’s playgrounds usable? (If people are using it, it is usable. If no one is using it, consider whether it is forbidden, or abandoned.)
Answer – “Yes”
42. How many types of Kid’s playgrounds equipment are present? (e.g., swings, seesaws, slides, trampolines, carousels, rock climbing, etc.)
Answer – “≥10”
43. Are there any guidance or warning signs for Kid’s playground users?
Answer – “Yes”
44. Are the Kid’s playgrounds in good condition? (Consider the cleanliness, management and safety)
Answer – “A lot”

Running track

45. Are there any tracks specifically for running?
Answer – “No”
46. Are there any obstacles on the running tracks that prevent you from moving on?
Answer – “Not applicable”
47. Are the running tracks in good condition? (Consider the cleanliness, surface and management)
Answer – “Not applicable”

Fitness area

48. Are there any fitness areas in the park?
Answer – “Yes”
49. Are there any guidance or warning signs for fitness area users?
Answer – “No”
50. Are the fitness areas in good condition? (Consider the cleanliness,

management and safety)
Answer – “Some”

Public /Green space

51. Are there any public /green spaces in the park? (It is a space where people can freely access and conduct activities.)

Answer – “Yes”

52. Are the surface of the public /green spaces flat?

Answer – “A lot”

53. Are there any guidance or warning signs for public /green spaces users?

Answer – “Yes

54. Are the public/green spaces in good condition? (Consider the cleanliness, safety, and maintenance)

Answer – “A lot”

Facilities and Amenities

Path

55. Are there any walking paths in the park?

Answer – “Yes”

56. Are the paths with the appropriate width for walking?

Answer – “Yes”

57. Are there any obstacles on the path?

Answer – “None”

58. Are the paths sloping?

Answer – “None”

59. Are the paths in good condition? (Consider the cleanliness, maintenance and surface)

Answer – “A lot”

Public toilet

60. Are there any public toilets in the park?

Answer – “Yes”

61. Are there any amenities offered in the toilets? (e.g., hand soap, hand towels, mirrors, toilet paper, etc.)

Answer – “A lot”

62. Are the public toilets in good condition? (Consider the cleanliness, maintenance and safety)

Answer – “A lot”

Shelter and pavilion

63. Are there any shelters and pavilions in the park?

Answer – “Yes”

64. Are there any benches to rest under the shelters and pavilions?

Answer – “Yes”

65. Are the shelters and pavilions in good condition? (Consider the cleanliness, safety and maintenance.)

Answer – “A lot”

Drinking fountain

66. Are there any drinking fountains in the park?

Answer – “No”

67. Are the drinking fountains usable? (If people are using it, it's usable. If no one is using it, consider whether it is vacated, destroyed, or abandoned)

Answer – “Not applicable”

Bench

68. Are there any benches to rest in the park? (Excluding all benches in themed areas such as sports courts, or playgrounds)

Answer – “Yes”

69. Are there any tree shades or coverage on the benches?

Answer – “Some”

70. Are there any pleasant views while resting on the benches?

Answer – “A lot”

71. Are the benches in good condition? (Consider the cleanliness, maintenance and comfort.)

Answer – “Some”

Picnic table

72. Are there any picnic tables in the park?

Answer – “Yes”

73. Are there any tree shades or coverage on the picnic table?

Answer – “A lot”

74. Are the picnic tables in good condition? (Consider the cleanliness, maintenance and comfort.)

Answer – “A lot”

Trash can

75. Are there any trash cans in the park?
Answer – “Yes”
76. Are the trash cans easy to find in the park?
Answer – “Some”
77. Are the trash cans in good condition? (Consider the cleanliness, maintenance, and smell)
Answer – “Some”

Aesthetic

Vegetation and landscape

78. How many species of vegetation can you identify in the park? (Including flowers and plants)
Answer – “>30”
79. Has any vegetation and landscape in the park been uprooted or damaged?
Answer – “None”
80. Are the vegetation and landscape in good condition? (Consider the management, mow, and design)
Answer – “A lot”

Graffiti

81. Are there any graffiti (unauthorized writing or drawing on a public surface) in the park?
Answer – “No”

Litter

82. Are there any litter in the park? (Do not consider litter inside the trash cans).
Answer – “Some”

Safety

Lighting

83. Is there adequate lighting in the park? (e.g., lighting in the most part of the park)
Answer – “A lot”

Surveillance equipment

84. Is there surveillance equipment in the park?

Answer – “Yes”

Emergency device

85. Are there any emergency devices in the park? (e.g., fire alarms, fire hydrants, Automated External Defibrillators (AED), or health service stations)

Answer – “No”

86. Are the locations of any emergency equipment marked on the park map?

Answer – “Not applicable”

Warning sign

87. Are there any warning signs in the park? (e.g., no swimming, no smoking, and dogs are prohibited).

Answer – “Yes”

88. Are these warning signs clear?

Answer – “A lot”

Supplementary Materials S2: Numerical codebook derived from the TAPS items structure to guide domain-level score calculation

The Purpose of this Codebook

In this study, parks were audited using audiT tool for Activity-friendly Parks in denSe urban areas (TAPS). It consists of 74 items across 24 criteria and five domains to evaluate parks in dense urban area. The main purpose of this codebook was to make sure all domains were graded using the same scoring scale. Another important purpose was to explain how to calculate each domain score, because these scores were based on several audit items, not just one. For this reason, the codebook clearly described the steps needed to calculate each domain score.

The framework of the TAPS instrument

The TAPS instrument included 74 quantifiable items, which were answered using either a dichotomous format (“Yes”, “No”) or a three-point format (e.g. “None”, “Some”, “A lot”). These 74 items were organised into 24 criteria, which were further grouped into five domains: Surroundings and Accessibility, Activity Areas, Facilities and Amenities, Aesthetics, and Safety.

Steps for numerical coding

Step 1: Item coding

For audit items with dichotomous responses, “Yes” should be coded as 1 and “No” as 0. For items with a three-point response format, “None”, “Some”, and “A lot” should be

coded as 0, 0.5, and 1, respectively. In some cases, the three-point options differ (e.g., “≤5”, “6–9”, “≥10”), but the corresponding codes remain 0, 0.5, and 1.

Examples:

Are there any signs showing the location or directions to the park?

☐ No ☐ Yes

If the response is “No”, the code should be assigned as 0; if the response is “Yes”, the code should be assigned as 1.

Are the entrance points attractive for residents?

☐ None ☐ Some ☐ A lot

If the response is “None”, the code should be assigned as 0; if the response is “Some”, the code should be assigned as 0.5; and if the response is “A lot”, the code should be assigned as 1.

How many entrance points are there to access the park smoothly?

☐ 1-2 ☐ 3-5 ☐ >6 (or park boundary is open)

If the response is “1–2”, the code should be assigned as 0; if the response is “3–5”, the code should be assigned as 0.5; and if the response is “>6 (or park boundary is open)”, the code should be assigned as 1.

However, some items are negatively coded; therefore, their coding needs to be reversed accordingly.

Examples:

Are there any obstacles on the sidewalk/bike lane that prevent you from moving on?

No Yes

If the response is “No”, the code should be assigned as 1; if the response is “Yes”, the code should be assigned as 0.

Has any vegetation and landscape in the park been uprooted or damaged?

None Some A lot

If the response is “None”, the code should be assigned as 1; if the response is “Some”, the code should be assigned as 0.5; and if the response is “A lot”, the code should be assigned as 0.

How long is the walking or cycling time from the public transport station to the nearest park entrance point (min)?

☐ <5 ☐ 6-10 ☐ 11-15

If the response is “<5”, the code should be assigned as 1; if the response is “6-10”, the code should be assigned as 0.5; and if the response is “11-15”, the code should be

assigned as 0.

Especially, item 18 was the only case in which the coding scale was modified. Auditors were asked to select up to three types of sports courts available in the park. Responses were coded as 0, 0.33, 0.67, and 1 for zero, one, two, and three selected court types, respectively.

Example:

What sports courts are in the park?

Cricket pitch   Football ground   Badminton court

Volleyball court   Basketball court   Handball court   Tennis court

Table tennis court   Baseball court   Other_________

Responses were coded as follows: No sports courts selected → 0; One court type selected → 0.33; Two court types selected → 0.67; Three court types selected → 1

<u>Step 2: Calculate the score for the criteria</u>

After each single item is coded, the scores for the 24 criteria in the TAPS instrument can be calculated separately. Each criterion consists of several items. The score for a given criterion should be computed using the following formula:

Score for a criterion=Total score of all items under that criterion ÷ Number of items included in that criterion

Example:

Traffic safety

1. Are there any crosswalks on the road to access the park?

☐ No ☑ Yes

2. Are there any traffic lights on the crosswalk?

☑ No ☐ Yes

3. Are there any signs of a speed limit on the roads to access the park?

☑ No ☐ Yes

4. Is the traffic situation around the park generally safe for residents?

☐ None ☐ Some ☑ A lot

Total score of all items under this criterion = 1 + 0 + 0 + 1 = 2

Number of items included = 4

Score for the criterion "Traffic Safety" = 2 ÷ 4 = 0.5

The calculation method described above was applied to all criteria except for the criterion "**Sport Court**", which includes items 18-25. A maximum of three sport court types can be selected in item 18, and auditors can answer items 19–25 separately.

In this criterion:

Item 18 represents the quantitative aspect (how many types of sports courts exist).

Items 19–25 represent the qualitative aspect (details of each court).

The score for the criterion “Sport Court” should be calculated as:

[Quantitative aspect score + (Qualitative aspect score ÷ 21)] ÷ 2

Here, 21 represents the total number of questions derived from items 19–25. Each of the seven items could be answered up to three times.

Example:

18. What types of sports courts are in the park?

☑ *Cricket pitch*	☑ *Football ground*	□ *Badminton court*
□ *Volleyball court*	□ *Basketball court*	□ *Handball court*
□ *Tennis court*	□ *Table tennis court*	□ *Baseball court*

Other__________________

Court 1. __ Cricket pitch ____	*Court 2. __Football ground____*	*Court 3. ___________*

19. Are the sports courts usable?

□ *No*	☑ *Yes*	□ *No*	☑ *Yes*	□ *No*	□ *Yes*

20. Are the sports courts free to use?

□ *No*	☑ *Yes*	□ *No*	☑ *Yes*	□ *No*	□ *Yes*

21. Are there any guidance or warning signs for sports court users?

☑ *No*	□ *Yes*	□ *No*	☑ *Yes*	□ *No*	□ *Yes*

22. Are there any benches for resting?

☑ *No*	□ *Yes*	☑ *No*	□ *Yes*	□ *No*	□ *Yes*

23. Are there any shade areas or shelters near the sports courts?

□ *No*	☑ *Yes*	□ *No*	☑ *Yes*	□ *No*	□ *Yes*

24. Are the sports courts fenced?

□ *No*	☑ *Yes*	□ *No*	☑ *Yes*	□ *No*	□ *Yes*

25. Are the sports courts in good condition?

□ *Not*	☑ *Some*	□ *A lot*	□ *Not*	□ *Some*	☑ *A lot*	□ *Not*	□ *Some*	□ *A lot*

Quantitative aspect score: The score of item 18 = 0.67

In item 18, two types of sports courts (Cricket pitch and Football ground) were selected; therefore, item 18 received a score of 0.67.

Total score of the qualitative aspect:

Cricket pitch quality + Football ground quality

= (1 + 1 + 0 + 0 + 1 + 1 + 0.5) + (1 + 1 + 1 + 0 + 1 + 1 + 1)

= 10.5

Final calculation: Score for the criterion "Sport Court" = [0.67 + (10.5 ÷ 21)] ÷ 2 = 0.585

Step 3 (Final step): Calculate the score for domains

After the scores for all 24 criteria are calculated, the score for each corresponding domain is then computed using the following formula:

Score for a domain = Total score of all criteria under that domain ÷ Number of criteria included in that domain

Example:

Domain: Aesthetic

Vegetation and landscape

64. How many species of vegetation can you identify in the park? (Including flowers and plants)

□ *<10* ☑ *11-30* □ *>30*

65. Has any vegetation and landscape in the park been uprooted or damaged?

☑ *None* □ *Some* □ *A lot*

66. Are the vegetation and landscape in good condition? (Consider the management, mow, and design)

□ *None* □ *Some* ☑ *A lot*

Graffiti

67. Are there any graffiti (unauthorized writing or drawing on a public surface) in the park?

☑ *No* ◻ *Yes*

Litter

68. Are there any litter in the park? (Do not consider litter inside the trash cans).

◻ *None* ☑ *Some* ◻ *A lot*

Total score of all criteria under that domain = score of "vegetation and landscape" + score of "graffiti" + score of "litter" = [(0.5 + 1 + 1) ÷ 3] + (1 ÷ 1) + (0.5 ÷ 1) = 2.33

Number of criteria included in that domain = 3

Final calculation: Score for the domain "Aesthetic" = 2.33 ÷ 3 = 0.78

Supplementary Materials S3. Comparison of ChatGPT-5 and Expert Scores Using Wilcoxon Signed-Rank Test.

Criteria	Group	Sig. (Two-tailed)
Accuracy in park surroundings and accessibility	ChatGPT-5 - Expert	0.10
Accuracy in activity area	ChatGPT-5 - Expert	0.06
Accuracy in facilities and amenities	ChatGPT-5 - Expert	0.64
Accuracy in aesthetics	ChatGPT-5 - Expert	0.66
Accuracy in safety	ChatGPT-5 - Expert	0.10
Accuracy [a]	ChatGPT-5 - Expert	0.14
Domain coverage	ChatGPT-5 - Expert	0.38
Strengths count	ChatGPT-5 - Expert	0.70
Weaknesses count	ChatGPT-5 - Expert	0.37
Recommendations match	ChatGPT-5 - Expert	$p<0.05$
Recommendations mismatch	ChatGPT-5 - Expert	0.15
User coverage	ChatGPT-5 - Expert	0.80
Issue resolution rate	ChatGPT-5 - Expert	0.09

[a] Accuracy represents the average accuracy score across the five park domains; $p < 0.05$
